# Direct force measurement and loading on developing tissues in intact avian embryos

**DOI:** 10.1242/dev.201054

**Published:** 2023-05-04

**Authors:** Chon U. Chan, Fengzhu Xiong, Arthur Michaut, Joana M. N. Vidigueira, Olivier Pourquié, L. Mahadevan

**Affiliations:** ^1^ John A. Paulson School of Engineering and Applied Sciences, Harvard University, Cambridge, MA 02138, USA; ^2^ Institute of Molecular and Cell Biology, A*STAR, Singapore 138673; ^3^ Department of Pathology, Brigham Women's Hospital and Department of Genetics, Harvard Medical School, Boston, MA 02115, USA; ^4^ Wellcome Trust/CRUK Gurdon Institute, University of Cambridge, Cambridge CB2 1QN, UK

**Keywords:** Tissue forces, Avian embryo, Feedback-control, Loading, Body axis

## Abstract

Developmental morphogenesis is driven by tissue stresses acting on tissue rheology. Direct measurements of forces in small tissues (100 µm-1 mm) *in situ*, such as in early embryos, require high spatial precision and minimal invasiveness. Here, we introduce a control-based approach, tissue force microscopy (TiFM), that integrates a mechanical cantilever probe and live imaging with closed-loop feedback control of mechanical loading in early chicken embryos. By testing previously qualitatively characterized force-producing tissues in the elongating body axis, we show that TiFM quantitatively captures stress dynamics with high sensitivity. TiFM also provides the means to apply stable, minimally invasive and physiologically relevant loads to drive tissue deformation and to follow the resulting morphogenetic progression associated with large-scale cell movements. Together, TiFM allows us to control tissue force measurement and manipulation in small developing embryos, and promises to contribute to the quantitative understanding of complex multi-tissue mechanics during development.

## Teaser

**Highlighted Article:** A new feedback-controlled precision technique to measure and alter tissue forces in early avian embryos.

## INTRODUCTION

During the development of a multicellular organism, cell behaviours collectively generate tissue forces and often alter tissue mechanical properties. These changes drive tissue deformations and lead to functional shapes and patterns. Understanding the dynamics and regulation of these mechanical factors is essential for creating accurate models of tissue morphogenesis and their eventual control, questions that span the entire range from basic to applied developmental biology, e.g. organoid engineering. Tissue size remains a major constraint for mechanical studies of early animal embryos, where the fundamental body plan and a variety of distinctly structured and shaped tissues form rapidly at the small scale of 100 µm (Mongera et al., 2019). At these developmental stages, tissues are very soft and produce small stresses. A number of *in vivo* approaches have been developed to address this challenge in early embryos. These include classic embryology methods, such as surgical cutting (Schoenwolf and Smith, 1990; Xiong et al., 2020), which allows inference of tissue mechanical interactions; microaspiration (Kim et al., 2020), which measures tissue mechanical properties; cantilever beams and fibres (Chevalier et al., 2016; Hara et al., 2013) and embedding gels (Zhou et al., 2009), which measure tissue stresses; and laser ablation (Hutson et al., 2009), which assesses tissue tension, among others (Campàs, 2016). Emerging (in the sense that they are more recently applied to early embryos) techniques incorporating precision engineering methods also show great promise, particularly in the measurement of tissue mechanical properties in intact embryos (or large explants). Examples include imaging-based methods, such as optical coherence elastography (OCE) (Mulligan et al., 2016) and Brillouin microscopy (Prevedel et al., 2019) operating with certain mechanical models. Actuator-based approaches, such as magnetic droplets (Serwane et al., 2017), atomic force microscopy (AFM) (Barriga et al., 2018) and related microindenters (Marrese et al., 2020), and optically trapped nanoparticles (Dzementsei et al., 2018), offer direct mapping of the spatial-temporal mechanical heterogeneity of tissues and can also be used to introduce a controllable load to tissues *in situ*.

Using embedded soft alginate gels, our previous work (Xiong et al., 2020) detected a pushing force from the axial tissues (neural tube and the notochord) of early chicken embryos (HH8-12; Hamburger and Hamilton, 1951) that drives body elongation and cell movement near the posterior progenitor domain. Given that only very soft alginate gels show visible deformation, it is likely that this pushing force was very small. However, it was not possible to use the gels for accurate quantification of the force as they were heterogenous, irregularly deformed and likely change their mechanical properties in the chemical environment of the developing embryo. Furthermore, as the size of the gels were relatively large (dozens of cell diameters) and stayed in the tissue for a long period of time (Xiong et al., 2020), the deformation induced by them at the local embedding site could alter the cell organization and tissue mechanics of the normal tissue environment, making tissue force quantification inaccurate. To minimize the tissue impact of force sensors, ultra-thin retrievable probes can be used, which reduce the size and duration of contact required for the measurements. Here, we present a new system using a micro-cantilever deflection-based approach (Hara et al., 2013), which uses a beam/needle that is bent when one end is held still and the other end is under a load. By combining modern cantilevers, live imaging and tracking, and electronic sensing in a programmed feedback loop, we show that it is possible to construct a system capable of dynamic force measurement. Here, we present the design and validation results of the system, which we termed Tissue Force Microscopy (TiFM), and discuss its application to characterizing and altering the active forces in live avian embryos.

## RESULTS AND DISCUSSION

**Fig. 1.**
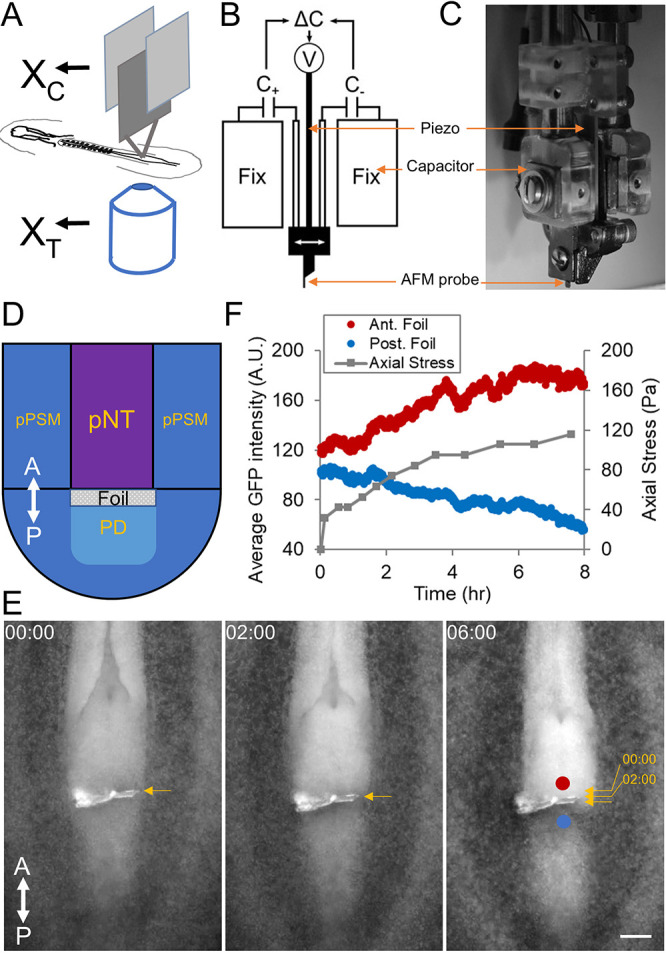
**Tissue force microscopy (TiFM) to measure the axial elongation force.** (A) The concept of TiFM. The design takes advantage of the flatness of the early avian embryo. X_C_ is the holder ‘chip’ position measured by the capacitors, X_T_ is the probe ‘tip’ position measured by the microscope. The difference between them is a measure of the deflection of the cantilever beam. (B,C) Probe holder and capacitors. Two capacitor plates and the piezo are integrated for position control and measurement against two fixed capacitor plates. C, capacitance; V, voltage. (C) A side photo of the assembled probe holder. (D) Diagram of the axial elongation stress measurement. This is a dorsal view of the tail end of the embryo, as seen in E; the probe enters from the ventral side. A-P-labelled double-headed arrows indicate the antero-posterior axis. pPSM, posterior presomitic mesoderm; pNT, posterior neural tube; PD, progenitor domain. Cells from the PD enter the U-shaped PSM under the pushing forces from the pNT and notochord (Xiong et al., 2020). (E) Movie frames at the times indicated (h:min). Foil movement under the elongation force. Arrows indicate the small displacements of the foil (overlaid on the third image). The foil depth is ∼200 µm. Blue and red circles indicate the regions of interest for fluorescence intensity measurements. This is a GFP embryo. Neural tube folds can be seen to be closing and narrowing. Representative of five similar experiments. (F) Axial elongation stress and cell density approximated by fluorescence intensity. The stress is calculated from the displacement, the cantilever spring constant (0.2 N/m) and the cross-sectional area of the foil. Scale bar: 100 µm.

The key for accuracy of a cantilever is the precision of the deflection measurement, which comes from the positional difference between the clamped end and the loaded end. The bigger the deflection, the better the signal-to-noise ratio. To match the sensitivity required for the small stresses produced by soft body axis tissues in the early chicken embryo, we used commercially available atomic force microscope (AFM) silicon-nitrate probes as our cantilevers. These probes can have low stiffness constants to the order of 0.01 N/m (10 nN/µm to put in the small tissue perspective). In contrast to the tapping mechanism in AFM surface imaging, we position these thin (∼1 µm) cantilevers vertically to allow direct insertion into the tissue, with or without modifications to the tip. In the case of measuring the axial pushing force, because the tissue cross-section is much larger than that of the cantilever tip, we glued a tailored piece of aluminium foil (200 µm square, ∼15 µm thick) to the tip (Michaut, 2018), which fully blocks the elongating neural tube and notochord in a HH11 chicken embryo upon insertion. The embryo (prepared using the *ex ovo* EC culture protocol on a piece of windowed filter paper; Chapman et al., 2001) is mounted on a glass-bottomed dish and imaged from below (Fig. 1A, Fig. S1). The glass-bottomed dish contains a thin layer of culture gel to support the embryo, and the embryo is covered by a thin layer of phosphate-buffered saline (PBS) and mineral oil to prevent drying (Michaut, 2018). The whole stage is set in an environmental enclosure maintaining 37.5°C with heating fans. Embryos develop normally at a slightly lower rate for at least 6 h under these conditions, as assessed by somite formation and axis elongation (∼2 h per somite compared with the ∼1.5 h normal rate, ∼100 µm/h elongation speed compared with the ∼150 µm/h normal rate). Notably, the probe insertion site heals quickly after cantilever retraction and becomes barely distinguishable under 1 h with foil and in a few minutes without foil. The inverted microscope (10× objective) captures images of the tissue section where the probe tip/attached foil is in focus and sends them to the computer for real-time segmentation to measure the position of the tip.

To enable dynamic positioning of the cantilever, the chip holding the cantilevers is mounted on an electric piezo (Fig. S2). To enable precise measurement of the chip/piezo position, they are further flanked with a pair of capacitors (Fig. 1B,C). The capacitance difference between the pair is highly sensitive to the distance between the capacitor plates and therefore the movement of the piezo. Before loading the embryo, the chip position and the capacitance reading are first calibrated with the microscope to create a lookup function where capacitance difference is interpreted as chip position. This real-time position information can then be sent as feedback to the voltage controller connected to the piezo as a closed loop system (Fig. 1B-D, see Materials and Methods). Voltage can thus be adjusted automatically if any drift of the piezo and/or chip is detected, ensuring the stability of chip position. Extended imaging of the chip confirms that the feedback loop maintains stable chip positioning.

By taking the position differences between the foil (measured by the microscope) and the chip (measured by the capacitors) over time, and multiplying the cantilever spring constant (0.2 N/m) and dividing by the foil cross-sectional area, we found the initial stress [shortly after (under 30 min) probe insertion] to be ∼40 Pa for axial elongation and the stalling stress in the longer term (>5 h) to be ∼100 Pa (Fig. 1D-F). Cells are observed to accumulate anterior to the foil as the foil moves and eventually stalls (Movie 1). Posterior to the foil, the cell density markedly reduces (Fig. 1F). The stalling condition where large local tissue deformation has occurred does not represent the normal condition of the tissues but helps assess the force-producing capacity of the tissue. Foil alone directly connected to the holding chip does not show any stable displacement (Movie 2, Fig. S3A). These results are consistent with our previous gel deformation experiments to detect the axis elongation force (Xiong et al., 2020). To assess the invasiveness of the foil, which causes a significantly larger area of tissue damage than sharp AFM probes (100-400 µm wide and ∼15 µm thick, compared with 30-50 µm wide and ∼1 µm thick), we took advantage of the piezo driver to cut different-sized foil insertion wounds at the body axis end and followed them over time (Fig. S3B). Thin slits (<20 µm) that are typically left after stress measurements heal quickly (in under 1 h), suggesting minimal cellular changes at the wound site (such as epithelialization of the exposed cells, which builds tension that prevents wound closure, a common occurrence in tissue microsurgeries that cause mechanical artifacts) and minimal long-term effects on the tissue area.

**Fig. 2.**
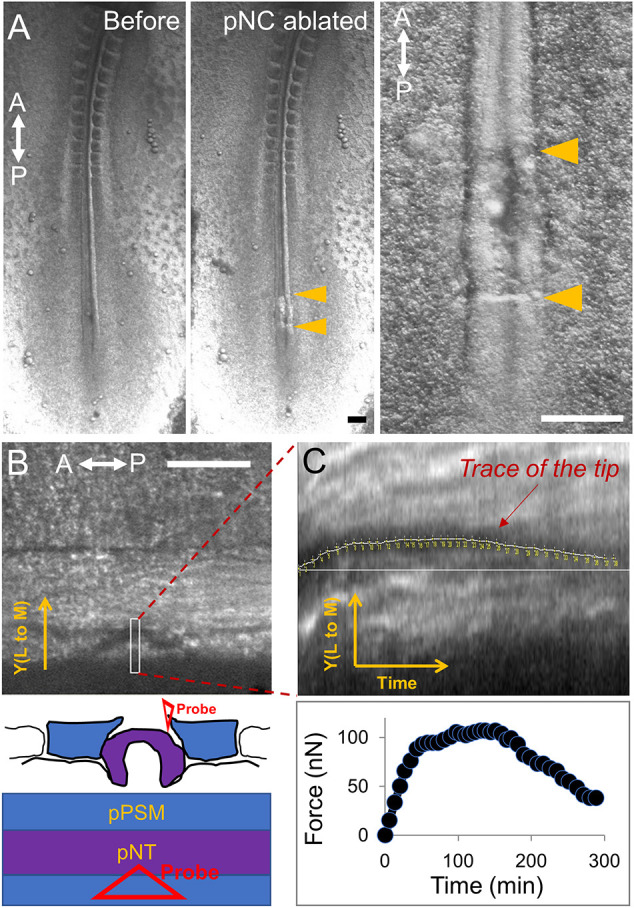
**Measurement of the posterior presomitic mesoderm compression.** (A) Ventral view of a HH11 avian embryo undergoing posterior notochord (pNC) ablation to reveal the medial surface of the pPSM. Arrowheads indicate the anterior and posterior borders of the surgical window. Neural tube that is more dorsal remains intact and is in the view. Representative of three similar experiments. A-P-labelled double-headed arrows indicate the antero-posterior axis. Scale bars: 200 µm. (B) Dorsal side view of the region in A now under the TiFM. As indicated in the diagram, a soft triangular probe (0.01 N/m) is now inserted medially by the pPSM boundary and the shadow of the triangle tip is visible. The pPSM tissue is known to expand into this area after the pNC is ablated. pPSM, posterior presomitic mesoderm; pNT, posterior neural tube. Scale bar: 100 µm. (C) Trace of the probe tip shows its deflection over time and translates into the lateral to medial force generated by the pPSM. The force quickly stalls around 100 nN and dissipates after a few hours. The estimated depth of the probe is ∼30 µm and contact surface size with the probe is in the order of 10^3^-10^4^ µm^2^, predicting a pPSM stress in the range of 10-100 Pa.

We next measured the stress produced by the posterior presomitic mesoderm (pPSM) flanking the body axis and known to be drivers of elongation (Bénazéraf et al., 2010). In this case, the tissue convergence speed (∼15 µm/h) is much slower than axis elongation (∼150 µm/h). In addition, because the notochord normally also undergoes tissue-autonomous convergence and resists PSM deformation, inserting the probe between the notochord and pPSM does not distinguish the source of the detected force. To ensure that we can test whether pPSM generates a stress, we positioned the probe next to the pPSM after surgically removing a region of the posterior notochord (Fig. 2A). Unlike the anterior PSM (aPSM) or somites, pPSM tissue is known to undergo expansion and will fill this opening after surgery (Xiong et al., 2020). A small stress in the range of 10-100 Pa is detected that gradually dissipates over several hours (Fig. 2B,C). This is consistent with our previous observations that the pPSM compresses axial tissues and that the compression disappears at the level of the differentiating aPSM (Xiong et al., 2020). Our system thus enables direct quantitative (at the precision of an order of magnitude in this particular case) confirmation of the small forces generated by the tissues of the elongating chicken body axis.

**Fig. 3.**
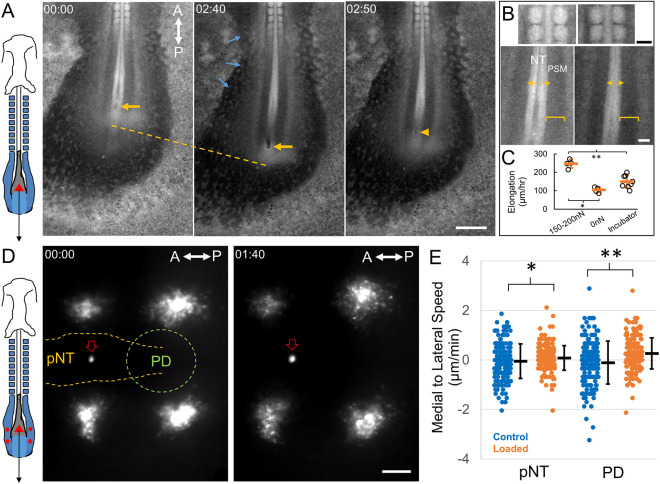
**Tissue and cell dynamics after mechanical load.** (A) Schematic and images from a loaded avian embryo. The red triangle in the diagram indicates the probe tip. The black arrow indicates the direction of the force. Posterior body axis and extra-embryonic regions (including zona opaca) are visible in the images. This is a GFP embryo. Yellow arrows indicate the probe location. Yellow arrowhead shows the wound immediately after probe retraction. Yellow dashed line measures elongation. Blue arrows indicate expansion of the zona opaca towards the embryo under load. Time stamps are in h:min. Images are representative of five similar experiments. A-P-labelled double-headed arrows indicate the antero-posterior axis. Scale bar: 500 µm. (B) Magnified views of body axis tissues under load, comparing 00:00 and 02:40 timepoints from A. Somites and anterior neural tube are largely unchanged. Posterior neural tube markedly narrows while the PSM narrows only a little. NT, neural tube; PSM, pre-somitic mesoderm. Scale bars: 100 µm. (C) Physiological loading increases elongation speed. Loaded samples are under 150-200 nN (*n*=4). 0 nN indicates no load control on the TiFM (*n*=4). Incubator control includes samples not on the TiFM mounting environment (*n*=8), which develop faster. Data are mean±s.d. *P<0.001 and **P<0.001 (unpaired two-tailed *t*-tests). (D) Schematic and images from a loaded and DiI-labelled embryo. The probe tip is fluorescent in the red channel, similar to DiI (red arrow in the images). The red triangle in the diagram indicates the probe tip. The black arrow indicates the direction of the force. Red circles mark the DiI injection sites in the pPSM corresponding to the clusters of cells on the image. pNT, posterior neural tube; PD, progenitor domain. Two clusters are at the same anterior-posterior level as the pNT and two are at the same level as the PD. Some of the DiI-labelled cells in these movies can be tracked to analyse cell movements. Time stamps are h:min. Images are representative of four similar experiments. Scale bar: 100 µm. (E) Medial to lateral speeds of cells measured from tracks at the pNT and PD levels, respectively. Each speed measurement is calculated from the displacement of a cell over a 5 min interval. Loading causes cells to move more laterally, on average. Data are mean±s.e.m. *P=0.032 and **P<0.001 (unpaired two-tailed *t*-tests).

To perform controlled mechanical perturbations, we used the feedback loop to move the piezo and/or chip to maintain a constant deflection by comparing with the tip position obtained with live segmentation of the tip images. This enables a sustained constant force to be applied to the tissue through the cantilever tip. Using this system, we loaded an anterior-to-posterior steady pulling force (150-200 nN) on the axial tissues, which at the same time is also a pushing force on the posterior progenitor domain (Fig. 3A). The embryos show accelerated elongation under this load and surrounding tissues exhibit differently patterned deformations (Fig. 3A-C, Movies 3 and 4). For example, the neural folds showed clear fastened convergence and closure under loading while PSM elongated without pronounced width change (Fig. 3B). It is also notable that, despite being under a strong load that doubled elongation speed (Fig. 3C), the insertion location and surrounding tissues remained intact and no tearing was observed, and the insertion wound quickly disappeared after probe retraction (Movie 4). We labelled cell clusters in the pPSM and followed their movements by cell tracking (Fig. 3D). The stress loading caused the cell cluster to move more laterally, following the ‘U’-shaped trajectory from the progenitor domain to the pPSM (Fig. 3E), consistent with more invasive approaches such as a magnetic pin that produces excess stresses beyond the physiological range (Xiong et al., 2020). These data together show that, in an intact embryo, tissue stresses exerted at one location have wide impacts through inter-tissue connections and alter cell behaviours at a distance, highlighting the importance of integrated multi-tissue models in developmental morphogenesis.

We name the close-loop electro-mechanical system, including the vertical cantilever, the piezo, the capacitors, live imaging and incubation described here, Tissue Force Microscopy (TiFM, Fig. S1). TiFM theoretically reaches a sensitivity of 1 nN (limited by the resolution and accuracy of tip imaging and tracking) with the present hardware and has 3D coverage at ∼20 µm spatial resolution (typical widths of the probe tip) parallel to the stress and 1-30 µm along the direction of the stress (depending on how the tip is modified, e.g. fluorescent dye, foil, etc.). The main measurement error terms arise as the probe interfaces with the complex and heterogenous embryonic tissues. For example, as the deflection increases, the deviation from the vertical of the contact angle to the tissue becomes more significant. Imperfections during foil preparation can add errors to the tissue contact area and increase tissue damage. In thicker tissues, probe tip tracking is more error-prone due to reduced contrast in the images, although this could be improved by modified tips such as those with glue and fluorescent dyes, which create a trackable pattern (with trade-off of spatial resolution). These factors should be considered when estimating the stress measured or inflicted. For a detailed discussion of sources of errors and how to estimate/control them, see Materials and Methods section ‘Sources and considerations of measurement errors’.

Using TiFM, a stress measurement against a deforming tissue takes 10-30 min to reach stalling conditions (when further deflection of the probe by the tissue becomes minimal). This may take longer when the contact area is larger, as in the case of a foil-probe construct, and thus the probe needs to be retracted to minimize long-term invasiveness. The sharp tip creates little tissue damage (such as tearing) even with a strong load. These features are advantageous as the tissues are measured more closely to their native state with smaller and shortened local deformations. By applying a well-controlled stress close to the endogenous force of the tissue *in vivo*, downstream cellular responses such as gene expression changes can now be studied in more physiologically relevant ranges and with reduced experimental noise that is often difficult to achieve with mechanical perturbations. We believe that the path is now open to combine TiFM loading with genetic probes to allow molecular force reporters to be calibrated (Kim et al., 2015). The difficulty of using inverted microscopy with a thin flat sample like the early chicken embryo can be overcome with self-detecting probes or alternative deflection detectors, such as an interferometer. Future work will aim to improve the automation and throughput of TiFM, and to expand its applications to rheological measurements and other model systems. Altogether, TiFM shows promise by adding to the expanding toolbox (Campàs, 2016) for understanding the physical mechanisms of morphogenesis and the quantitative engineering of development in small tissues.

## MATERIALS AND METHODS

### Eggs and embryo preparation

Wild-type chicken (*Gallus gallus*) eggs were supplied by Charles River Laboratories and Medeggs (Fakenham, UK). Tg(CAG-GFP) (McGrew et al., 2008) chicken eggs were provided by Clemson University (SC, USA; originally by the University of Edinburgh). Eggs were kept in a monitored 15°C fridge for storage and in egg incubators at 37.5°C and ∼60% humidity for incubation. HH stage 10-12 embryos were used. The early stage embryos are used under a tissue protocol and do not require an animal protocol according to institution guidelines. To obtain the embryos for TiFM measurements, eggs were incubated for ∼40 h before opening for the EC culture (Chapman et al., 2001). The EC culture uses 2 cm×2 cm pieces of filter paper (Whatman) with two adjacent 0.5 cm holes in the centre. Eggs were opened into a petri dish and the thick albumen on the top that covers the embryo and the vitelline membrane was swept aside gently with small filter paper pieces using a tweezer. The filter paper with holes was then lowered to attach to the vitelline membrane in such a way that embryos were visible through the hole (body axis aligned to the long axis of the hole). The vitelline membrane was then cut around the filter paper to release the embryo. The filter culture embryo was then rinsed in PBS to remove excess yolk. The cleaned embryo was then placed on a 3.5 cm petri dish containing 2 ml of culture gel made according to the following formula (per 100 ml of culture gel): part A, 50 ml albumin (beaten for 15 min) then supplemented with 0.8 ml 20% D-glucose (Sigma); part B, 0.3 g BactoAgar (Sigma) dissolved in 50 ml water in a microwave then supplemented with 1.23 ml 5 M NaCl. Part A was warmed and part B cooled to 55°C in a water bath and mixed thoroughly then added to petri dishes (2 ml each) before gelation. The embryo cultures were then stored in a slide box with wet paper towels in the incubator. In experiments where some tissue areas and cells are labelled with DiI, the DiI was injected with a sharp-tipped glass needle by mouth pipetting from the ventral side of the embryo. The stock solution of 2.5 mg/ml DiI in ethanol was diluted in PBS to 0.5 mg/ml before injection. At the sample-loading step of the TiFM procedure (see below), two embryos were taken and transferred to a pre-warmed glass-bottomed imaging dish (MatTek) covered with 200 µl of culture gel. A second piece of filter paper was then added to prevent the embryo from detaching and floating once it was submerged. Pre-warmed PBS was then added to cover the embryos, followed by one or two drops of mineral oil – enough to spread out and cover the surface. One embryo was subjected to TiFM measurements and/or loading while the other served as a control inside the incubation chamber on the microscope.

### Design and operation of the TiFM

A working TiFM can be assembled with the list of required equipment and components below. Design considerations are described and the components used in this study are listed, but it is not necessary to acquire the same components. The construction of the probe holder and incubation chamber would depend on the configuration of the base microscope that is used. Similarly, existing microscope software can be incorporated into the operation procedure. Users with electrical engineering and programming experience are required for the assembly and maintenance of the system.

#### Required equipment and components

##### Microscope

To construct a TiFM system, an inverted microscope with *xy* stage control and *z* focus control is required. We used the Zeiss Axio Observer base (top modules including the TL illumination and condenser were removed). A low-magnification objective of 2.5-5× is required for sample positioning and a 10× objective is required for image data streaming.

##### Camera

Owing to the lack of TL illumination and the size and close proximity of the probe holder to the sample, side LEDs were included to compensate for the lack of light on the sample. A sensitive, fast camera is required to provide high-resolution streaming of the probe tip in the tissue, which is essential for real-time feedback control of the force. The temporal and spatial resolution limit of the system is set by the camera and imaging protocol (described in more detail in the ‘Operation’ section). We used a Ximea USB camera (MQ042MG-CM).

##### Custom probe holder and capacitors

A stable, controllable probe needs to be installed on a reliable micromanipulator and/or stage as a holder with minimal drift over time. We used a World Precision Instruments WPI M3301R Manual Micromanipulator and a Newport 9062-XYZ-M stage. The stage was fixed to optical rails and beams (Thorlabs) onto the microscope base, forming an overhang on top of the sample stage. We 3D printed two-part plastic holders where the sample side has a slot for the chip of AFM probes and the piezo side has a slot for the insertion of the mobile end of the piezo (Fig. S2), and two slots on either side for the mobile copper capacitor plates. The two parts were tightened with screws to allow piezo and probe exchanges. The static end of the piezo was inserted into another printed holder that connected to a cage holding the fixed capacitor plates. The capacitors flanked the piezo, the positioning of which affected the capacitance difference (Fig. 1B-C), allowing a calibration of capacitance to holder position at the beginning of an experiment (described in more detail in the ‘Operation’ section).

##### Voltage controller and piezo

A programmable voltage controller is required to drive the piezo. We used a custom-built controller integrating a low and high power source; however, commercial controllers (such as Thorlabs, MDT694B) would also work. The controller needs to be able to adjust voltage output quickly and accurately during live measurements to enable the feedback control. The piezos we used are the ceramic piezoelectric benders from Thorlabs (PB4NB2W). Components in the section ‘Custom probe holder and capacitor’ should be designed in accordance with the type of the piezo and working range required.

##### AFM probes

We used smooth (no tip modifications) silicon nitrate AFM probes from Bruker (MLCT-O10) and NanoAndMore (AIO-AL-TL) with spring constants ranging from 0.01 to 0.2 N/m. Comparable probes would be feasible to use. It is advantageous to use probes with a larger length as they can reach deeper points in the embryo.

##### Environmental chamber

The embryo requires proper temperature and humidity to develop normally. Common lab and microscope facility environments have a low humidity and room temperature around 25°C. We used a layer of mineral oil to reduce evaporation but this is not the optimal method, as long-term survival of the embryo is affected under this condition. Environment chambers where humidity can be maintained at a high level while the electronics still function would be desirable. Alternatively, oxygenating the culture media that submerges the embryo may also be effective. We used a custom laser-cut cardboard box to enclose the holder and the sample stage, and heating fans integrated with temperature sensors to maintain 37.5°C. Commercially available environmental chambers would also work but customization (e.g. additional holes) is needed to allow the installation of the probe holder and the electrical wires.

#### Optional components

##### Microcontroller

Because multiple data streams (images, capacitance, voltage, temperature, etc.) flow through the system, it is advantageous to organize them under an integrated controller to align data onto the same time axis. For force measurement and loading, small time differences in data streams do not cause a major issue because the probe and sample move slowly and errors average out through the feedback over time. However, for other measurements that can potentially be made using TiFM, such as oscillatory rheology, time axis alignment is crucial. In these situations, a master clock is used to trigger the camera and capacitance readings for synchronization. We used a Teensy microcontroller to link different parts of the system and interface with Matlab (Mathworks) on the computer.

##### Illumination modules

Having good contrast on the probe tip and embryo tissues is important for the precision of tip positioning and position measurement via image segmentation. The overhanging holder including the capacitors and the piezo form an occlusion for overhead TL illumination. Small LEDs can be installed on the holder to directly illuminate the sample. We used side LEDs that are fixed on the sample stage. The LEDs can be triggered by the camera or the microscope. We also used the fluorescence module of the Zeiss scope to provide RL illumination of the GFP transgenic embryos, and the probe tip that can be coloured using Quantum dots glued on using epoxy, mixing dyes such as DiI with the epoxy or simply taking advantage of the autofluorescence of epoxy.

#### Software

To measure and control forces, the locations of the chip and the tip are streamed in real time, and the chip location can be altered by piezo movement. Therefore, the main functions to achieve with the software are to send and receive the chip and/or piezo position (i.e. voltage), to program imaging, to receive images and to obtain tip position. We used Matlab (MathWorks) to create the user interface that plots the serial data via a USB link to the microcontroller, and to run the image segmentation. For the objective and shutter control, we used Micromanager (Edelstein et al., 2014). A Teensy program that integrates the data streams is uploaded to the microcontroller before the start of experiments.

#### Operation

##### Testing and calibration

An assembled TiFM system needs to be tested and calibrated before loading actual samples. This step ensures the system functions properly and produces necessary parameters and data for the sample measurements. First, holder stability must be tested for the desired duration of the experiment. Without samples, the microscope should be used to take a timelapse of the overhang holder (1) without and (2) with the voltage controller on, and (3) with the capacitor-voltage controller feedback loop on where a set capacitance is sent by the software. For a stable system, all timelapse results should show minimal movement of the probe, but the system is usable if (1) is stable and (3) achieves correction for drifts in (2). (1) tests the stability of the scaffold, such as the rails, columns and the micromanipulator. (2) tests the stability of the piezo and voltage controller output. (3) tests the capacitor positioning system and the feedback. Second, the correlation between capacitance differences and the position of the holder needs to be established by driving the piezo across its dynamics range while capturing holder movement with timelapse imaging. These data serve as a lookup table that links capacitance reading to holder position, which will be used in sample measurements where the holder position can no longer be measured by imaging because of sample obstruction.

##### Sample loading and probe insertion

Because the sample will result in light obstruction and scattering, the microscope view of the probe holder and tip will be blurry, making the probe insertion process difficult to see from the camera. Therefore, the *xy* position of the probe should be marked on the field of view before sample loading. The probe holder is then raised in *z* (without touching its *xy* control) in order to make room for the sample. After the sample dish (see ‘Embryo preparation’ section for the protocol of readying a chicken embryo for TiFM) is in place, the desired tissue location can be aligned to the mark using the sample stage so that the probe tip will enter the correct location once it is lowered again. Once the probe enters the liquid layers, care must be taken to lower the probe further to the desired tissue depth slowly without overshooting, which could cause tip breakage and/or tissue damage. The entrance of the probe and/or foil into the tissue is usually smooth because of their sharpness. Light conditions may make it difficult to see the location of the probe tip. It is advisable to adjust the objective focus around the sample plane to find the probe tip. Once the probe is in the proper tissue location and in focus, camera and lighting settings can be further adjusted to ensure good contrast images at a fast rate (low exposure time).

##### Force measurement and loading

To measure tissue forces and/or stresses, the probe should be inserted to block the direction of tissue movement. If the desired measurement cross-section of the tissue is larger than the probe, a piece of aluminium foil can be attached to the probe tip via epoxy. We cut the foil pieces using a micromanipulator with a blade under a dissecting microscope to obtain rectangular pieces of 100-400 µm. To determine the contact area between the tissue and the probe in order to calculate the stress (rather than just the total force), the insertion depth is measured by the *z* positioning system of the microscope. Using the Zeiss Axio Observer as an example: first, the objective position is recorded from the *z*-controller screen when the focus is on the surface of the tissue (e.g. endoderm for a dorsally mounted embryo, as in Fig. 1E); second, the objective is moved (lowered) to focus on the vicinity of the tissue layer of desired insertion depth (e.g. dorsal edge of the neural plate, as in Fig. 1E); third, the probe (with or without foil) is inserted until the tip or edge of the foil is in focus at the desired depth, some minor adjustment of probe depth and/or focus might be performed for best focus and contrast, then the *z* position of the objective is recorded again. Comparing the recorded objective *z* positions yields the insertion depth. Using the insertion depth and known shapes of the probes and/or foils and the tissue, the tissue contact area can be estimated. To measure the stalling stress, once the tip and/or foil is in position, the capacitance should be fixed through the feedback loop to maintain the position of the holder. Timelapse imaging of the tissue area and the tip and/or foil displacement then indicates the force. The displacement will increase quickly then slowly and finally stall as morphogenesis is stalled by the probe. To load the tissue with a specific force, the force value will be evaluated against the current probe location and capacitance reading, and an adjustment of capacitance target (i.e. holder position) will be sent to the voltage controller. With continued imaging and segmentation during the experiment, the feedback loop maintains a dynamically stable difference between the holder and the probe tip (i.e. cantilever deflection and force). After completion of measurements, the probe should be washed in deionized water by dipping to prevent damage from culture gel, albumen and salt crystals after drying.

##### Sources and considerations of measurement errors

The working principle of the cantilever (with force constant k) method requires accurate measurements of the location of the holder (X_C_) and the tip (X_T_). For dynamic measurement and feedback control, these two measurements should also be synchronized in time. Therefore, factors that introduce inaccuracy for positional measurements and synchronization will bring error terms to the force measurements. In addition, because tissue stress (σ) is biologically more meaningful to calculate than the detected or inflicted force (F), which varies with tissue contact area (A), the requirement of contact area estimation raises additional error terms [σ=k(X_T_-X_C_)/A]. For this current version of the TiFM, the largest error term is associated with the lowest resolution aspect of the system, which is the tracking of the tip position (hardware limited to ∼200 frame/second and ∼0.5 µm pixel sizes). The capacitors and piezos are subject to fluctuations in the hardware, such as from the voltage controller, but the sampling rate is higher and the errors are of a much smaller order of magnitude. Moreover, during force control, although there is a delay between imaging and segmentation of the tip to the action of voltage adjustment that may cause a force error, such errors will average out quickly over time through the feedback. Therefore, the main error considerations focus on the spatial accuracy of X_T_ and the estimation of A. Segmentation and tracking of the tip (X_T_) without an embryo sample (e.g. in air or water) produce high accuracy to the camera resolution. Errors increase as the imaging depth through the tissue (D_I_) increases, which deteriorates the tip image contrast and signal-to-noise ratio. This can be mimicked by imaging the probe movement behind increasingly thicker gels that scatter the light from the tip. In the case of the embryo, the scattering will be additionally complex due to tissue heterogeneity. The exact positional uncertainty of the tip imaged through thick tissues depends on the conditions under which the images are obtained and should be taken into consideration when designing experiments. Using fluorescently labelled probes and surgically removing some tissues to image through are both effective ways of controlling this error. For the estimation of A, the insertion angle (θ_I_), depth (D) and features of the probe tip (e.g. dye, foil) should be considered. Taking the axis elongation force for example, the posterior body axis growth is largely horizontal during the stages concerned, therefore a vertical insertion of probe is desirable. The insertion angle (θ_I_) is usually not perfectly vertical but can be adjusted by rotation of the mounting arm while moving the focal plane along the probe length (L) between the probe tip and base to minimize the on-camera horizontal movement. This can reach a sin(θ_I_)<0.05 for a L=200 µm probe. The accuracy of depth of insertion (D), as obtained from the protocol described in the section ‘Force measurement and loading’ depends on the recognition of focal planes of tissue surfaces and probe tips by the user, and can in practice have ±20 µm uncertainties that lead to uncertainties in contact area estimation. Depending on the type of tip, foils can have a 10-20% uncertainty in A, while narrow probes can be accurate only by an order of magnitude in terms of stress estimation under an error range of ±20 µm in *z* (e.g. Fig. 2C). Other factors include the quality of the foil surface and edges, where some curvature may make the effective A smaller than that of a flat foil. Effective ways in controlling the errors for A include higher precision manufacturing of foils or other thinner materials (such as mica); transgenic fluorescent embryos that enhance the recognition of tissue layers and/or surfaces through focusing on cell layers. As an example, a well-preadjusted probe (θ_I_<15°) and a thin sample tissue location (such as the pPSM where both D_I_ and D<100 µm) enables stress measurements by TiFM with a maximum 20% uncertainty term with a foil-probe construct (100 µm wide), giving a high degree of confidence in the quantitative characterization of tissue forces.

### Data analysis

Movies were analysed in Fiji (ImageJ). Probe and/or foil displacement was measured by object tracking in the intensity-time plot. The fluorescence intensities (as a proxy to cell density) were measured by drawing a region of interest before and after the foil. DiI-labelled cells were tracked with the Manual Tracking plug-in. Tracking results and measurements were processed in Matlab (Mathworks) with custom scripts and plotted with Excel (Microsoft).

## Supplementary Material

10.1242/develop.201054_sup1 Supplementary information
