# Supplementary material for: Direct force measurement and loading on developing tissues in intact avian embryos
Source: Development. 2023 May 4;150(9):dev201054. doi: 10.1242/dev.201054 (PMC10259510; doi:10.1242/dev.201054)
Supplement: Supplementary information [file develop-150-201054-s1.pdf]

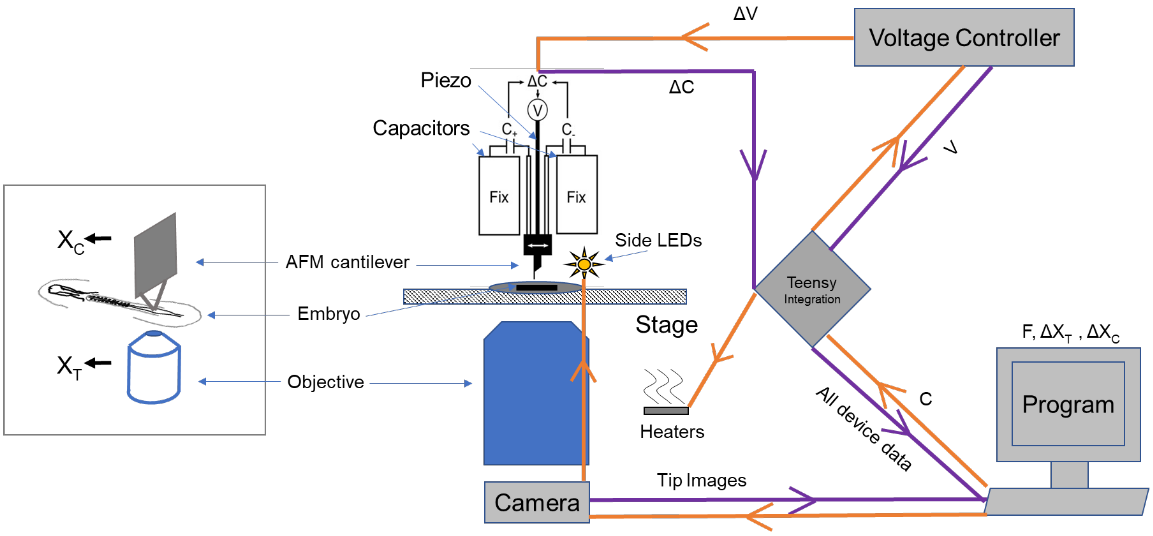

Fig. S1. Diagram of the TiFM system

The left image is the conceptual design related to the actual system on the right. Purple arrows indicate data flow and orange arrows indicate command signals.

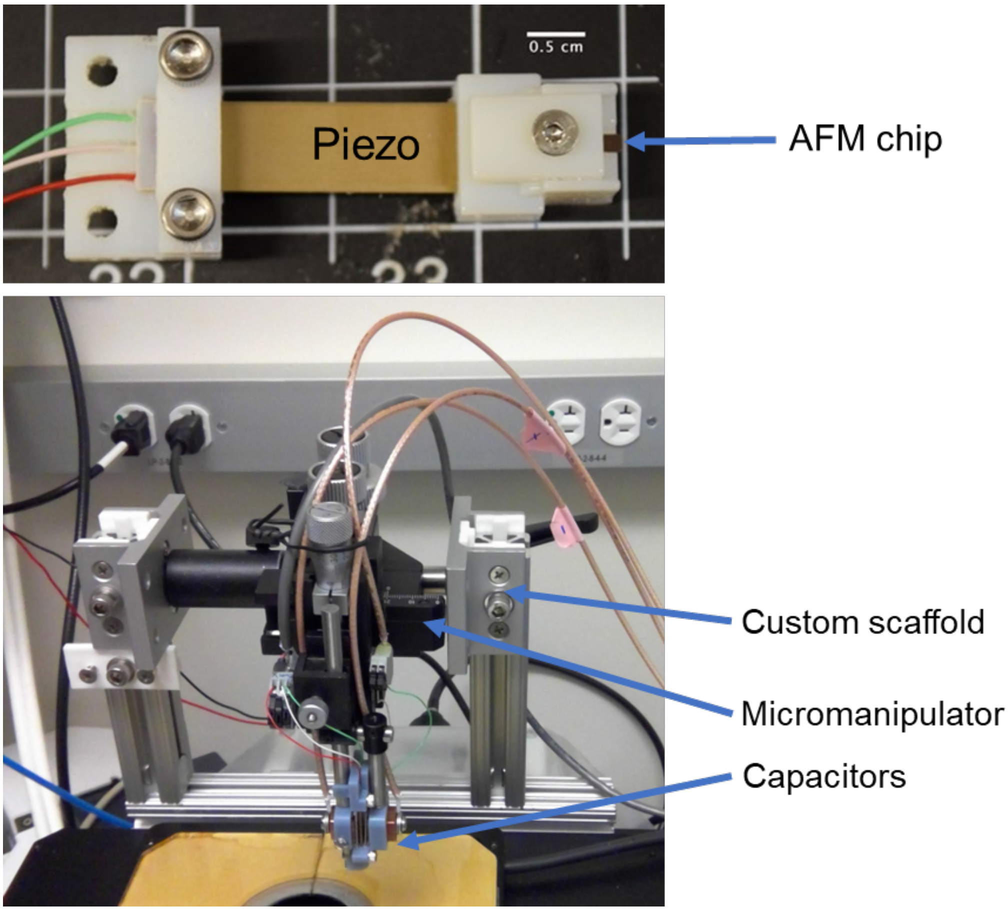

Fig. S2. Custom printed/built parts of the TiFM system

Top image shows the 3D-printed plastic piezo holders. Bottom image shows the overhang scaffold. These designs are flexible with the actual scope and piezo used.

Development • Supplementary information

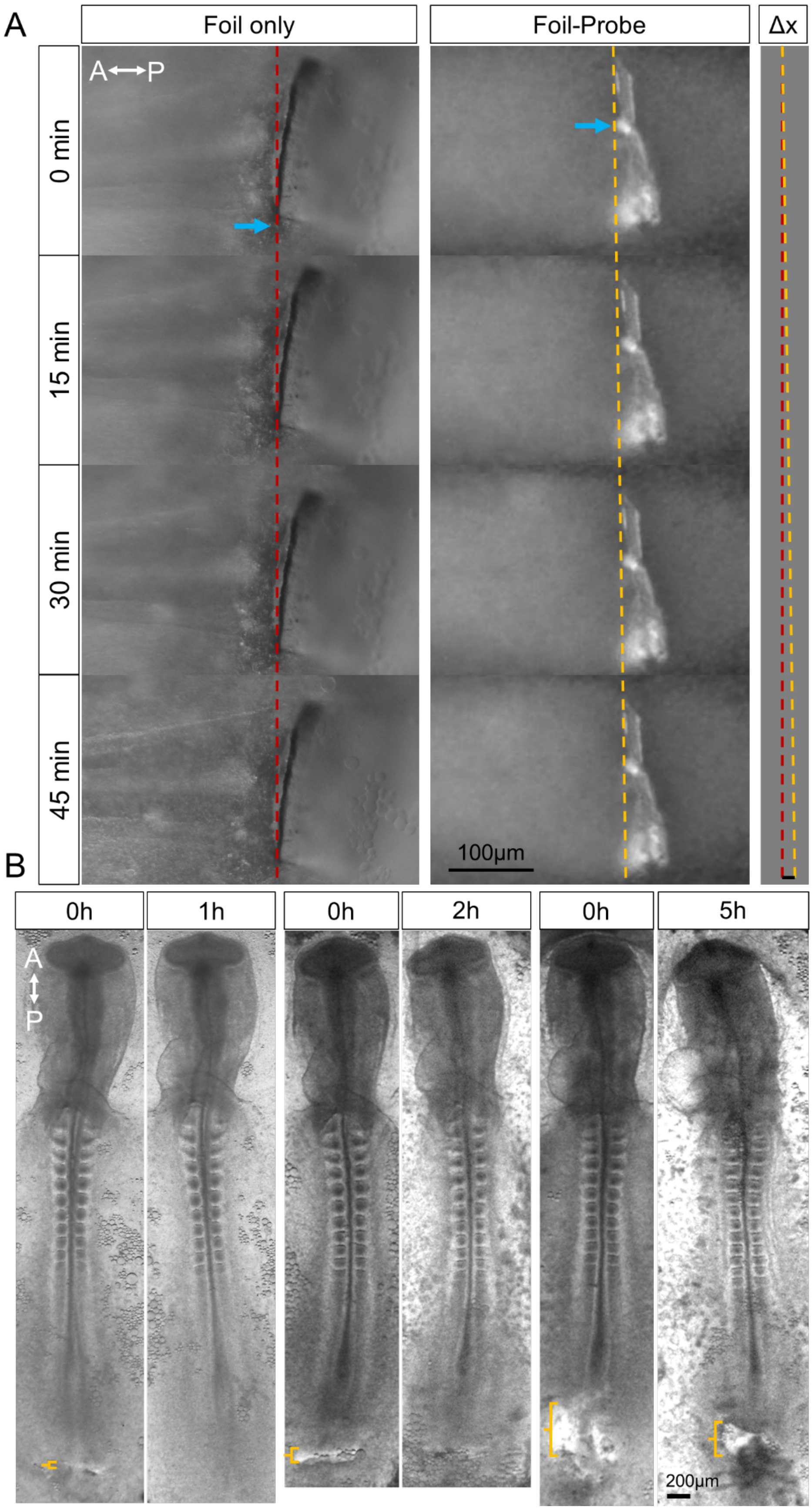

Fig. S3. Controls for foil insertion experiments

(A) Tissue forces are insufficient to bend aluminium foils. A stripe of aluminium foil tailored to a ~400µm (L) x ~200µm (W) stripe was glued directly to the holder chip

Development • Supplementary information

rather than the cantilever probe. Tracking of this foil (blue arrows indicate a high-contrast point of the foil that was tracked) shows that it remains static, while the foil attached to the probe (from Fig. 1E) shows a displacement (Δx) of ~17μm. The force constant of the foils (k) can be estimated using the following information: the thickness ~20μm (t), Young's modulus of aluminium ~70GPa (E), and the second moment of area of rectangular beams $I=Wt^3/12$. Here $k=3EI/L^3 \approx 500$ N/m in the direction of the force under a free-end load condition (the value would be higher for the actual condition). This is significantly higher than the cantilever probes used (<1 N/m) in the experiments.
(B) Tissue invasiveness of the foil experiments as evaluated by wound healing. The end of the body axis was actuated with a foil to create different sized wounds. For a slit-like wound the tissue heals under 1hr, as the wound widens, healing becomes slower and/or incomplete over a longer duration when the wounds are too large. For stress measurements, the wounds are slit-like and heal quickly. Yellow brackets mark the width of the wound after foil extraction along the AP axis.

Development • Supplementary information

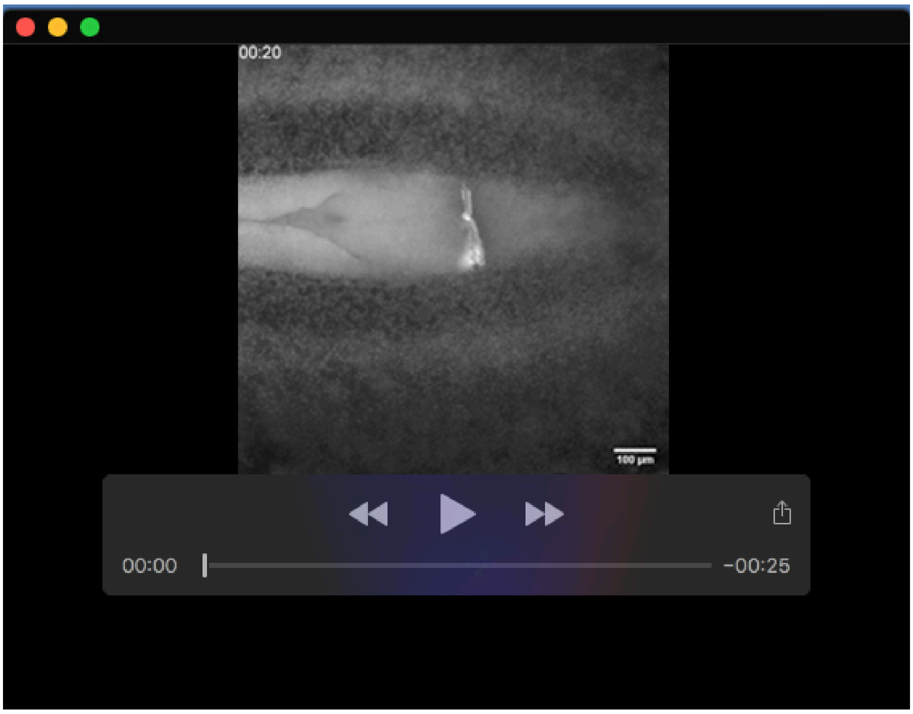

Movie 1. Measuring axis elongation stress with TiFM

Time stamps are hh:mm. This is a dorsal view of the tail end of a GFP embryo, anterior to the left. A small displacement of the foil is visible, providing information for inferring the stress. Refer to text and Fig. S3 for further details.

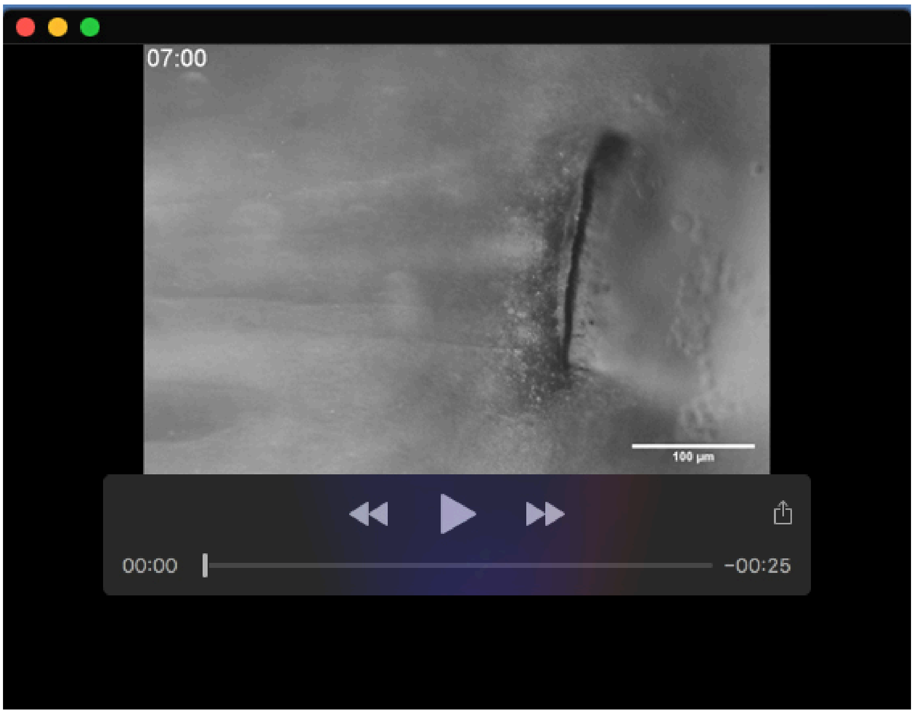

Movie 2. Foil-only control for axis elongation stress with TiFM

Time stamps are mm:ss. This is a dorsal view of the tail end of a WT embryo, anterior to the left. No stable displacement of the foil is observed (small short-term vibrations may be observed due to environmental noise). Refer to text and Fig. S3 for further details.

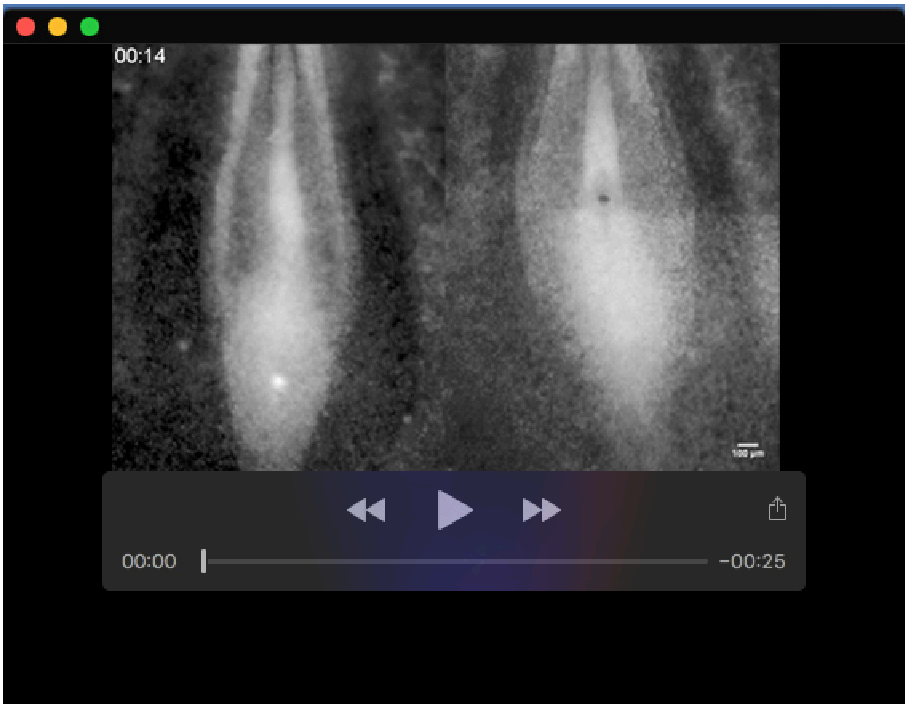

Movie 3. Force loading on a live embryo
Time stamps are hh:mm. Two GFP embryos, a control (left) and a loaded one (right) are shown, anterior to the top. Probe tip (modified with epoxy) is visible as a bright sphere with a dark bar in the center in the loaded embryo. The loaded embryo elongates at a faster rate. Refer to text for further details.

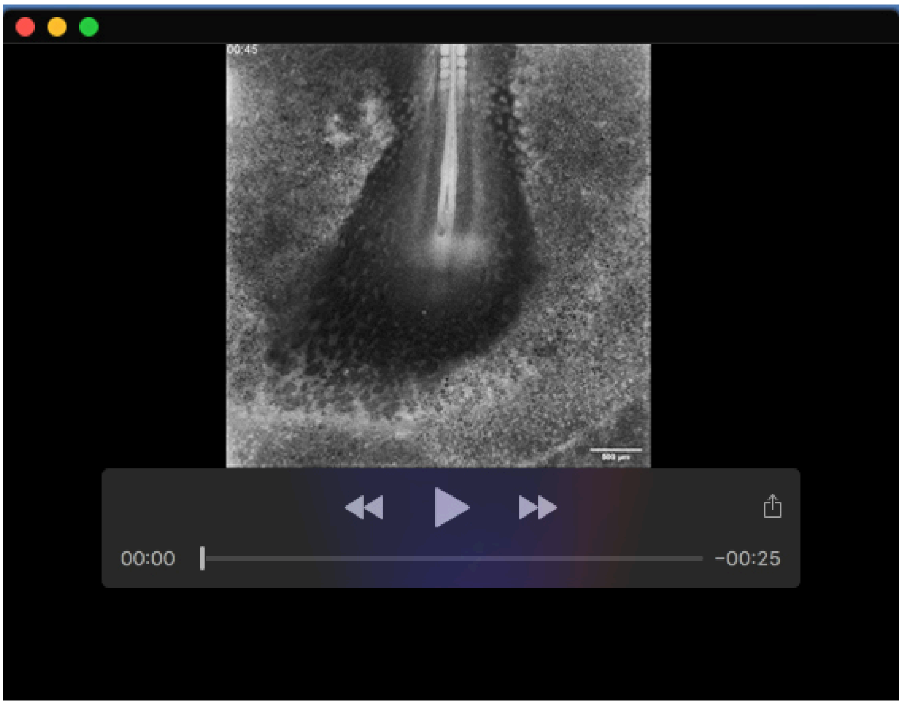

Movie 4. Patterned tissue deformation on a loaded embryo
Time stamps are hh:mm. This GFP embryo was loaded at 200nN and then unloaded. Anterior to the top. Probe tip is visible at the tail-end midline as a dark dot. Refer to text and Fig. 3A for further details.

Development • Supplementary information
