# Supplementary material for: Direct force measurement and loading on developing tissues in intact avian embryos
Source: Development. 2023 May 4;150(9):dev201054. doi: 10.1242/dev.201054 (PMC10259510; doi:10.1242/dev.201054)
Supplement: Supplementary file 1 [file develop-150-201054_review_history.pdf]

Direct force measurement and loading on developing tissues in intact avian embryos

Chon U. Chan, Fengzhu Xiong, Arthur Michaut, Joana M. N. Vidigueira, Olivier Pourquié and L. Mahadevan

DOI: 10.1242/dev.201054

Editor: Thomas Lecuit

Review timeline

Original submission:	20 June 2022
Editorial decision:	23 August 2022
First revision received:	15 February 2023
Editorial decision:	24 March 2023
Second revision received:	27 March 2023
Accepted:	6 April 2023

Original submission

First decision letter

MS ID#: DEVELOP/2022/201054

MS TITLE: Direct Force Measurement and Loading on Developing Tissues in Intact Avian Embryos

AUTHORS: Chon U Chan, Fengzhu Xiong, Arthur Michaut, Olivier Pourquie, and L Mahadevan

I sincerely apologize for the delay before I could come back to you. I have now received the reports of two referees on the above manuscript, and have reached a decision. The referees' comments are appended below, or you can access them online: please go to BenchPress and click on the 'Manuscripts with Decisions' queue in the Author Area.

As you will see, the referees express strong interest in your work, but one of the referees expresses some significant criticisms and recommend a substantial revision of your manuscript. If you are able to revise the manuscript along the lines suggested, which may involve further experiments, I will be happy receive a revised version of the manuscript. Your revised paper will be re-reviewed by one or more of the original referees, and acceptance of your manuscript will depend on your addressing satisfactorily the reviewers' major concerns. Please also note that Development will normally permit only one round of major revision. If it would be helpful, you are welcome to contact us to discuss your revision in greater detail. Please send us a point-by-point response indicating your plans for addressing the refereeâ€™s comments, and we will look over this and provide further guidance.

Please attend to all of the reviewers' comments and ensure that you clearly highlight all changes made in the revised manuscript. Please avoid using 'Tracked changes' in Word files as these are lost in PDF conversion. I should be grateful if you would also provide a point-by-point response detailing how you have dealt with the points raised by the reviewers in the 'Response to Reviewers' box. If you do not agree with any of their criticisms or suggestions please explain clearly why this is so.

Reviewer 1

Advance summary and potential significance to field

© 2023. Published by The Company of Biologists under the terms of the Creative Commons Attribution License (https://creativecommons.org/licenses/by/4.0/).

In this manuscript Chan et al., outline a new technique to directly assay the forces exerted by flat tissues in vivo such as chicken embryos. The system which they call Tissue Force Microscopy, consists of a cantilever mounted on a chip connected to a piezo, which detects the location of the chip. This is then mounted on an inverted microscope, which can then be used to image the location of the cantilever as it is inserted into a tissue. The authors measure the deflection of the cantilever by comparing the position of the cantilever tip through imaging with the piezo detected chip position, allowing them to calculate the force or pressure exerted on the cantilever. As a proof of principle, the authors then attempt to quantify forces in the chick embryo during the elongation of axial tissues that they had previously described. They show an additional function where they are able to exert a constant force on the tissue through automatic detection of the cantilever position. This is sent back to the piezo which can automatically adjust the chip position to maintain a constant deflection and therefore constant force.

Comments for the author

While the technique described by the authors is interesting, there are unfortunately major issues with the manuscript. Overall the presentation of both the figures and text is not clear, and a more exhaustive list of issues may be provided after the following points are addressed.

- There is almost no introductory text and very little context is provided for the manuscript. How can the reader know the relevance of the device to the study of embryonic development?
- What is the stiffness of the foil that is inserted in the tissue? It should be demonstrated that the foil is much stiffer than the cantilever and that it cannot be deformed by the forces exerted by the tissues as this would affect the force that actually gets transduced to the cantilever. In Figure 1 it seems as though the foil is already misshapen potentially changing the area of the foil in contact with the tissue.
- It is referred to as a non-invasive technique, but rather invasive perturbations are used. Are they required to measure stresses exerted by the tissue? Is the system sensitive enough to detect forces in unperturbed tissues?
- 'Elongation forces' are mentioned in figure 1 but we are not sure that this is what is being measured because the pressure builds as cells build up on the anterior side of the foil over 4 hours. The authors refer to this as a 'stalling stress' in section 4.3. We think it should be referred to 'stalling stress' throughout the manuscript and not equated to the elongation forces exerted by the axial tissues.
- Convergent forces in figure 2 – ablation of the NC could artificially increase the forces exerted on the probe as medial PSM migration is enhanced to fill the open space. Therefore, the reported force is likely not simply the physiological level of force exerted by the tissue during elongation. To measure the physiological levels of force this should be done without ablation while inserting the probe at the PSM/NC boundary. More generally, the invasiveness of the device should not be underestimated in the manuscript since wound response can generate very significant forces within tissues.
- When no probe/foil construct is used, how sure are the authors about the area of the probe in contact with the tissue? How is this accurately determined?

How consistent is the depth at which the probe enters the tissue from one sample to the other? This will have a large effect on measured forces and should be correctly estimated and discussed.

- When discussing figure 3 and MovieS2, it is claimed that the loaded tissues elongate faster and 'differently patterned deformations' are formed, but no quantification is provided. The difference in the 'patterned deformations' are particularly unclear.
- Very little discussion on the error associated with the measurements and no equations are shown, making it hard to discern how much error could be associated with different aspects of the technique, e.g. the error introduced by changing the XY resolution through use of a fluorescent probe tip versus an unmodified tip. Since the authors claim repeatedly to be able to measure small forces in a physiological range, knowing the measurement errors and the resolution of the measurement is key to the presentation of this technique.
- Referring to the technique as "TFM" will inevitably result in confusion with Traction Force Microscopy, a very established technique in mechanobiology.

A different abbreviation should be considered.

Reviewer 2

Advance summary and potential significance to field

© 2023. Published by The Company of Biologists under the terms of the Creative Commons Attribution License (https://creativecommons.org/licenses/by/4.0/).

This is a concise paper but contains a new approach that enables quantification and manipulation of forces in living embryo. This sort of technical challenges are required and should be appreciated in the filed of developmental mechanobiology, especially if this instrument is applicable to other model animals' embryo.

Comments for the author

Measurement and manipulation of physical forces that are applied on living tissues are very difficult technically and the development of such methodologies are certainly essential for the advancement of mechanobiology that uses living embryos. This paper by Chan et al. reports a new technology taking advantage of high sensitivity of force measurement by AFM, combined with sophisticated electro-engineering employing piezo and capacitor. This technique has been shown to enable not only measurement of minute forces generated in tissues of living embryos but also application of forces on tissues at any given time and place of embryos. This is an important step toward the further understanding of the physiological significance of forces during development.

This reviewer raises several minor points for improvement.

1. TFM is the abbreviation commonly used for traction force microscopy in the field of mechanobiology and thus it may confuse readers and the field. This reviewer suggests an alternative term, for example, TiFM.
2. In the summary, the word “non-invasive” may be too exaggerated. This may depend on how invasion is defined and it may be true if the method is compared to other harsh operations previously reported, but insertion of cantilever may be considered to be an invasion as it should cause some mechanical influence on the surrounding cells. Therefore better wording, or elaboration of why this method is considered to be non-invasive, is recommended.
3. In Fig. 1E, the right most panel, the coloring of blue and red dots does not make sense. Are they reversed?
4. In all photos and movies, it is useful to indicate A (anterior) and P (posterior). For Fig. 2B, C, “Y (space)” is fine but additional labeling is helpful.
5. It is useful to provide the information of approximate depth of the force measurements (cantilever) from the tissue surface.

First revision

<u>Author response to reviewers' comments</u>

Reviewer 1 Advance Summary and Potential Significance to Field:

In this manuscript Chan et al., outline a new technique to directly assay the forces exerted by flat tissues in vivo such as chicken embryos. The system, which they call Tissue Force Microscopy, consists of a cantilever mounted on a chip connected to a piezo, which detects the location of the chip. This is then mounted on an inverted microscope, which can then be used to image the location of the cantilever as it is inserted into a tissue. The authors measure the deflection of the cantilever by comparing the position of the cantilever tip through imaging with the piezo detected chip position, allowing them to calculate the force or pressure exerted on the cantilever. As a proof of principle, the authors then attempt to quantify forces in the chick embryo during the elongation of axial tissues that they had previously described. They show an additional function where they are able to exert a constant force on the tissue through automatic detection of the cantilever position. This is sent back to the piezo which can automatically adjust the chip position to maintain a constant deflection and therefore constant force.

Reviewer 1 Comments for the Author:
While the technique described by the authors is interesting, there are unfortunately major issues with the manuscript. Overall the presentation of both the figures and text is not clear, and a more exhaustive list of issues may be provided after the following points are addressed.
- There is almost no introductory text and very little context is provided for the manuscript. How can the reader know the relevance of the device to the study of embryonic development?

© 2023. Published by The Company of Biologists under the terms of the Creative Commons Attribution License (https://creativecommons.org/licenses/by/4.0/).

Thanks. We agree wholeheartedly that readers should be well informed. We have now included an introduction to the broader context of tissue mechanics in development, available techniques and challenges, and how our method fits in.

The introduction now reads: "*During the development of a multicellular organism, cell behaviors collectively generate tissue forces and alter tissue mechanical properties. These changes drive tissue deformation towards functional patterns and shapes. Understanding the dynamics and regulation of these mechanical factors is essential for creating accurate models and controls of tissue morphogenesis in both basic and applied fields of developmental biology, for example organoid engineering. Tissue size remains a major constraint for mechanical studies of early animal embryos, where the fundamental body plan and a variety of distinctly structured and shaped tissues form rapidly at the small scale of 100μm (Mongera et al., 2019). At these developmental stages, tissues are very soft and produce small stresses. Currently available in vivo approaches suitable for these early embryos include classic embryology methods such as surgical cutting (Schoenwolf and Smith, 1990), cantilever beams and fibres (Hara et al., 2013; Chevalier et al., 2016), embedding gels (Zhou, Kim and Davidson, 2009), laser ablation (Hutson et al., 2009) and emerging (in the sense that they are more recently incorporated for embryos) techniques incorporating precision engineering methods such as magnetic droplets (Serwane et al., 2017), atomic force microscopy (AFM) (Barriga et al., 2018) and related microindenters (Marrese et al., 2020), Brillouin microscopy (Prevedel et al., 2019) and optically trapped nanoparticles (Dzementsei et al., 2018) among others (Campàs, 2016). These methods provide access to tissue mechanical properties at various resolution and coverage in intact embryos (or large explants), but have limited success in measuring bulk tissue stresses in situ.*

Using embedded soft alginate gels, we previously detected a pushing force from the axial tissues (neural tube and the notochord) of early chicken embryos (HH8-12, (Hamburger and Hamilton, 1951)) that drives body elongation and cell movement near the posterior progenitor domain (Xiong et al., 2020). This pushing force was estimated to be quite small as only very soft alginate gels show marked deformation. The gels were not suitable for accurate quantification of the force as they were heterogenous, irregularly deformed and might undergo mechanical property changes in the chemical environment of the developing embryo. Another general issue with large-size (several to dozens of cell diameters) embedded sensors/actuators is that they cause a large deformation at the local embedding site which could alter the cell organization and tissue mechanics of the normal tissue environment. One way to minimize the tissue impact of force sensors is to use ultra-thin, retrievable probes, which reduces the size and duration of contact required for the measurements. Here we present a new system taking a cantilever deflection approach(Hara et al., 2013), which utilizes a beam/needle that is bent when one end is held still and the other end is under a load. By combining modern cantilevers, live imaging and tracking, and electronic sensing in a programmed feedback loop, we constructed a system capable of dynamic force measurement and loading in live avian embryos. In this paper we present the design and validation results of the system, which we termed Tissue Force Microscopy (TiFM), and discuss the considerations in its applications."

- What is the stiffness of the foil that is inserted in the tissue? It should be demonstrated that the foil is much stiffer than the cantilever and that it cannot be deformed by the forces exerted by the tissues as this would affect the force that actually gets transduced to the cantilever. In Figure 1 it seems as though the foil is already misshapen potentially changing the area of the foil in contact with the tissue.

In the revision we include the following: 1. The stiffness of the foil calculated from its shape and manufacturer and literature info; 2. A control experiment using no cantilever but foil alone, showing no movement of inserted foil in the tissue. These results show that the foil is significantly stiffer than the cantilever and could not be bent by the tissue forces.

Briefly, the foil used in the experiments (length ~400μm x width ~200μm x thickness ~20μm) can be estimated to have a stiffness (defined here as deflection per unit force, to compare with the force constants of the cantilever beams) of k=3EI/L^3=3*7e10*2e-4*(2e-5)^3/12/(4e-4)^3=438 N/m in the direction of the force (using free-end load condition which produces the softest estimate) which is orders of magnitude larger than the cantilevers used (<1 N/m). Here E is the Young's modulus of aluminium (70GPa), I is the second moment of area, and L is the length of the foil.

© 2023. Published by The Company of Biologists under the terms of the Creative Commons Attribution License (https://creativecommons.org/licenses/by/4.0/).

In the new Figure S3A and Movie S2, the foil is seen to not move in the same tissue location without the cantilever.

The foils used in the experiments are cut by hand with a micromanipulator under a dissection scope. The edges are not always perfectly smooth. Several degrees of bending may exist across the foil as observed by the reviewer. This has been noted in the revision and the error introduced to contact area is discussed in the new measurement error discussion section.

• It is referred to as a non-invasive technique, but rather invasive perturbations are used. Are they required to measure stresses exerted by the tissue? Is the system sensitive enough to detect forces in unperturbed tissues?

Thanks. We agree with the reviewer that our technique, as a contact/insertion method, carries certain degree of invasiveness as compared to non-contact methods. However, while non-contact methods such as Brillouin microscopy are available to measure certain aspects of tissue mechanical properties, they are not able to directly measure tissue stresses. Our probe is less invasive in comparison to other available methods for stress measurement (e.g., embedding gels/droplets) for two main reasons: first, the probe has a minimized insertion footprint (a few microns thick, or in the case of using foils, 10-20microns thick); second, it is only needed to be inserted for the duration of measurement and can be retracted.

In the revision we added evaluation experiments to help readers judge the degree of invasiveness. In the new loading experiment added (Figure 3A and Movie S4), we show that the wound quickly closes and disappears within ~15 minutes after probe retraction, despite large deformation caused by the loading, no tissue tearing was observed.

The invasiveness is increased when foil is used. With foil, the open slit takes some time to heal. Taking advantage of our control of the piezo and subsequently the probe and foil, we actuated the end of the body axis to create different sized wounds. In the new Figure S3B we show results for the readers to evaluate the invasiveness of the foil. For a slit-like wound the tissue heals under 1hr, as the wound widens, healing becomes slower and not possible. These results suggest that temporary, thin wounds as generated in our experiments have a low level of long-term invasiveness, as the opening is small and can recover, compared to large implant methods.

In the revision we comprehensively described the invasiveness and put the technique in proper context. We also changed the text “non-invasive” to “minimally-invasive” in light of the evidence provided.

The resolution of TiFM is limited by the imaging resolution of the probe tip, both spatially and temporally. We estimate a resolution at the order of 1nN with the softest probes, which is well under the tissue forces detected. For example, the probe detects forces in endogenous locations without surgery, such as in the case of the elongating axis (Figures 1E-F). One challenge of measuring tissue stresses in situ is that neighboring tissues act in concert during development and each tissue contributes to the stress the probe experiences at the particular location. To quantify the contribution of the posterior PSM, we ablated the stiff medial notochord. This experiment, as the reviewer points out, introduces invasion to the tissue area and might cause deviation from the unperturbed conditions. However, this allows a test of the compressive stress from the PSM separate from other tissues. Inserting the probe between the notochord and PSM without the surgery will make it difficult to interpret the results, as the notochord could be generating a pulling or pushing stress on the probe at the same time.
Therefore, the accompanying surgery is introduced for experimental design reasons. Generally, as our method relies on measuring deflection of the probe, it will not be able to measure the stresses without surgery when two tissues that push against each other in equilibrium (no movement or slow movement, such as between notochord and PSM)

In the revision we included these further explanations of the rationale of performing surgeries.

• ‘Elongation forces’ are mentioned in figure 1 but we are not sure that this is what is being measured because the pressure builds as cells build up on the anterior side of the foil over 4 hours. The authors refer to this as a ‘stalling stress’ in section 4.3. We think it should be referred to

© 2023. Published by The Company of Biologists under the terms of the Creative Commons Attribution License (https://creativecommons.org/licenses/by/4.0/).

'stalling stress' throughout the manuscript and not equated to the elongation forces exerted by the axial tissues.

Thanks. Yes, the stalling stress is expected to be larger than the elongation stress as cells build up (which is no longer the initial endogenous condition). We observed a rapid increase of stress within several minutes of insertion which would be the closest measure of the endogenous elongation force. This has now been clarified throughout the manuscript.

- Convergent forces in figure 2 – ablation of the NC could artificially increase the forces exerted on the probe as medial PSM migration is enhanced to fill the open space. Therefore, the reported force is likely not simply the physiological level of force exerted by the tissue during elongation. To measure the physiological levels of force this should be done without ablation while inserting the probe at the PSM/NC boundary. More generally, the invasiveness of the device should not be underestimated in the manuscript since wound response can generate very significant forces within tissues.

One challenge of measuring tissue stresses in situ is that neighboring tissues act in concert during development and each tissue contributes to the stress the probe experiences at the particular location. In addition, as our measurement depends on a detectable amount of displacement of the probe, it would work best where significant tissue deformation is expected. In the case of posterior PSM and NC, it has been suggested that PSM has a tendency to expand while NC resists such expansion medially. In this scenario, inserting the probe between the NC and PSM without the surgery will make it difficult to interpret the results, as the NC could be resisting the probe movement at the same time from the other side. The result will be a net stress causing tissue convergence, which by itself is important to measure, but does not test the hypothesis of PSM active expansion and axial compression.

To isolate the contribution of the posterior PSM, we ablated the stiff medial notochord. This experiment, as the reviewer points out, introduces invasion to the tissue area such as wounding and might cause deviation from the unperturbed conditions. However, this allows a direct test of the compressive stress from the PSM separate from other tissues. It remains unclear whether this surgery would artificially increase or decrease the PSM stress. A future pressure sensor that works at a finer length scale will be required to detect the stress between the PSM/NC boundary without tissue surgery.

These points have been included in the description of this experiment in the revision for clarity.

- When no probe/foil construct is used, how sure are the authors about the area of the probe in contact with the tissue? How is this accurately determined? How consistent is the depth at which the probe enters the tissue from one sample to the other? This will have a large effect on measured forces and should be correctly estimated and discussed.

The contact area estimation contains the main error term. In the revision we provide a detailed description of determining probe depth and tissue contact area.

Specifically, section 4.3 of materials and methods now includes: "*To determine the contact area between the tissue and the probe in order to calculate the stress (rather than just the total force), the insertion depth is measured by the Z positioning system of the microscope. Using the Zeiss Axio Observer as an example: first, the objective position is recorded from the Z-controller screen when the focus is on the surface of the tissue (e.g., endoderm for a dorsally mounted embryo such as in Fig. 1E); second, the objective is moved (lowered) to focus on the vicinity of the tissue layer of desired insertion depth (e.g., dorsal edge of the neural plate as in Fig. 1E); third, the probe (with or without foil) is inserted until the tip or edge of the foil is in focus at the desired depth, some minor adjustment of probe depth and/or focus might be performed for best focus and contrast, then the Z position of the objective is recorded again. Comparing the recorded objective Z positions yields the insertion depth. Using the insertion depth and known shapes of the probes and/or foils and the tissue, the tissue contact area can be estimated.*"

The depth location that the tip/foil reaches is very consistent as we can rely on the tissue features that are in the same focal plane as the probe tip. Such features (e.g., widest edges of the

© 2023. Published by The Company of Biologists under the terms of the Creative Commons Attribution License (https://creativecommons.org/licenses/by/4.0/).

notochord, upper point of the neural folds, etc) are very similar between embryos of similar stages. These are further improved by the use of transgenic fluorescent embryos. Despite these, the contact area estimated for a narrow probe would still be significant when both the Depth and Width are small.

Factors that impact the contact area include the insertion angle of the probe/foil, shape of the probe/foil and its relation to the tissue shape. These are discussed in the new measurement error section 4.4. and considerations of experimental design to control these errors are offered.

Specifically, section 4.4 reads: "*The cantilever (with force constant k) method's working principle requires accurate measurements of the location of the holder (XC) and the tip (XT). For dynamical measurement and feedback control, these two measurements should also be synchronized in time. Therefore, factors that introduce inaccuracy for positional measurements and synchronization will bring error terms to the force measurements. In addition, because tissue stress (σ) is biologically more meaningful to calculate than the detected/inflicted force (F) which varies with tissue contact area (A), the requirement of contact area estimation raises additional error terms (σ=k(XT-XC)/A). For this current version of the TiFM, the largest error term is associated with the lowest resolution aspect of the system, which is the tracking of the tip position (hardware limited to ~200 frame/second and ~0.5μm pixel sizes). The capacitors and piezos are subject to fluctuations in the hardware such as from the voltage controller, but the sampling rate is higher and the errors are on a much smaller order of magnitude. Moreover, during force control, while there is a delay between imaging and segmentation of the tip to the action of voltage adjustment which may cause a force error, such errors will average out quickly over time through the feedback. Therefore the main error considerations focus on the spatial accuracy of XT and the estimation of A. Segmentation and tracking of the tip (XT) without embryo sample (e.g., in air or water) produce high accuracy to the camera resolution. Errors increase as the imaging depth through tissue (DI) increases which deteriorates the tip image contrast/signal-to-noise ratio. This can be mimicked by imaging the probe movement behind increasingly thicker gels that scatter the light from the tip. In the case of the embryo the scattering will be additionally complex due to tissue heterogeneity. The exact positional uncertainty of the tip imaged through thick tissues depends on the conditions under which the images are obtained and should be taken into consideration when designing experiments. Using fluorescently labelled probes and surgically removing some tissues to image through are both effective ways of controlling this error. For the estimation of A, the insertion angle (θI), depth (D) and features of the probe tip (e.g., dye, foil) should be considered. Taking the axis elongation force for example, the posterior body axis growth is largely horizontal during the stages concerned therefore a vertical insertion of probe is desirable. The insertion angle (θI) is usually not perfectly vertical but can be adjusted by rotation of the mounting arm while moving the focal plane along the probe length (L) between the probe tip and base to minimize the on-camera horizontal movement. This can reach a sin(θI)<0.05 for a L=200μm probe. The accuracy of depth of insertion (D) as obtained from the protocol described in 4.3 depends on the recognition of focal planes of tissue surfaces and probe tips by the user, and can in practice have ±20μm uncertainties which lead to uncertainties in contact area estimation. Depending on the type of tip, foils can have 10-20% uncertainty in A while narrow probes can only be accurate in the order of magnitude in terms of stress estimation under an error range of ±20μm in Z (e.g., Fig. 2C). Other factors include the quality of the foil surface and edges, where some curvature may make the effective A smaller than that of a flat foil. Effective ways in controlling the errors for A include: higher precision manufacturing of foils or other thinner materials (such as mica); transgenic fluorescent embryos which enhances the recognition of tissue layers/surfaces through focusing on cell layers. As an example, a well-preadjusted probe (θI<15°) and a thin sample tissue location (such as the pPSM where both DI and D<100μm) enables stress measurements by TiFM with a maximum 20% uncertainty term with a foil-probe construct (100μm wide), giving a high degree of confidence in the quantitative characterization of tissue forces.*"

- When discussing figure 3 and MovieS2, it is claimed that the loaded tissues elongate faster and 'differently patterned deformations' are formed, but no quantification is provided. The difference in the 'patterned deformations' are particularly unclear.

In the revision we provide new results (new Figure 3A) that capture the patterned deformations of

© 2023. Published by The Company of Biologists under the terms of the Creative Commons Attribution License (https://creativecommons.org/licenses/by/4.0/).

the embryo under load. These include the sped-up axis elongation and folding of the posterior neural tube, relatively-stable shapes of somites and the width of the presomitic mesoderm (PSM), and lateral to medial expansion of the extraembryonic area (area opaca). These results show that loading induced deformation is tissue dependent and suggests that the transmission of the loading force may depend on existing mechanical patterns of each tissue and their connections. These observations show promise of revealing intertissue mechanics of morphogenesis through controlled loading.

- Very little discussion on the error associated with the measurements and no equations are shown, making it hard to discern how much error could be associated with different aspects of the technique, e.g. the error introduced by changing the XY resolution through use of a fluorescent probe tip versus an unmodified tip. Since the authors claim repeatedly to be able to measure small forces in a physiological range, knowing the measurement errors and the resolution of the measurement is key to the presentation of this technique.

This is an important point. We have now included a dedicated discussion of the measurement errors, to make sure readers/users are aware of the practicalities and limitations. Errors from the hardware components are insignificant in comparison with errors that arise at the interface between the probe and the tissues during live acquisition. So we focus on the latter.

Specifically, the discussion reads: "*TiFM theoretically reaches a sensitivity of 1nN (limited by the resolution and accuracy of tip imaging and tracking) with the present hardware and has 3D coverage at ~20μm spatial resolution (typical widths of the probe tip) parallel to the stress and 1-30μm along the direction of the stress (depending on how the tip is modified, such as fluorescent dye, foil, etc). The main measurement error terms arise as the probe interfaces with the complex and heterogenous embryonic tissues. For example, as the deflection increases the deviation of contact angle to the tissue from vertical becomes more significant. Imperfections during foil preparation can add errors to the tissue contact area and increase tissue damage. In thicker tissues, probe tip tracking is more error-prone due to reduced contrast in the images, although this could be improved by modified tips such as those with glue and fluorescent dyes which create a trackable pattern (with trade-off of spatial resolution). These factors should be considered when estimating the stress measured or inflicted.*"

And section 4.4 of materials and methods reads: "*The cantilever (with force constant k) method's working principle requires accurate measurements of the location of the holder (XC) and the tip (XT). For dynamical measurement and feedback control, these two measurements should also be synchronized in time. Therefore, factors that introduce inaccuracy for positional measurements and synchronization will bring error terms to the force measurements. In addition, because tissue stress (σ) is biologically more meaningful to calculate than the detected/inflicted force (F) which varies with tissue contact area (A), the requirement of contact area estimation raises additional error terms (σ=k(XT-XC)/A). For this current version of the TiFM, the largest error term is associated with the lowest resolution aspect of the system, which is the tracking of the tip position (hardware limited to ~200 frame/second and ~0.5μm pixel sizes). The capacitors and piezos are subject to fluctuations in the hardware such as from the voltage controller, but the sampling rate is higher and the errors are on a much smaller order of magnitude. Moreover, during force control, while there is a delay between imaging and segmentation of the tip to the action of voltage adjustment which may cause a force error, such errors will average out quickly over time through the feedback. Therefore the main error considerations focus on the spatial accuracy of XT and the estimation of A. Segmentation and tracking of the tip (XT) without embryo sample (e.g., in air or water) produce high accuracy to the camera resolution. Errors increase as the imaging depth through tissue (DI) increases which deteriorates the tip image contrast/signal-to-noise ratio. This can be mimicked by imaging the probe movement behind increasingly thicker gels that scatter the light from the tip. In the case of the embryo the scattering will be additionally complex due to tissue heterogeneity. The exact positional uncertainty of the tip imaged through thick tissues depends on the conditions under which the images are obtained and should be taken into consideration when designing experiments. Using fluorescently labelled probes and surgically removing some tissues to image through are both effective ways of controlling this error. For the estimation of A, the insertion angle (θI), depth (D) and features of the probe tip (e.g., dye, foil) should be considered. Taking the axis elongation force for example, the posterior body axis growth is largely horizontal during the stages concerned therefore a*

© 2023. Published by The Company of Biologists under the terms of the Creative Commons Attribution License (https://creativecommons.org/licenses/by/4.0/).

vertical insertion of probe is desirable. The insertion angle (θI) is usually not perfectly vertical but can be adjusted by rotation of the mounting arm while moving the focal plane along the probe length (L) between the probe tip and base to minimize the on-camera horizontal movement. This can reach a sin(θI)<0.05 for a L=200μm probe. The accuracy of depth of insertion (D) as obtained from the protocol described in 4.3 depends on the recognition of focal planes of tissue surfaces and probe tips by the user, and can in practice have ±20μm uncertainties which lead to uncertainties in contact area estimation. Depending on the type of tip, foils can have 10-20% uncertainty in A while narrow probes can only be accurate in the order of magnitude in terms of stress estimation under an error range of ±20μm in Z (e.g., Fig. 2C). Other factors include the quality of the foil surface and edges, where some curvature may make the effective A smaller than that of a flat foil.

Effective ways in controlling the errors for A include: higher precision manufacturing of foils or other thinner materials (such as mica); transgenic fluorescent embryos which enhances the recognition of tissue layers/surfaces through focusing on cell layers. As an example, a well-preadjusted probe (θI<15°) and a thin sample tissue location (such as the pPSM where both DI and D<100μm) enables stress measurements by TiFM with a maximum 20% uncertainty term with a foil-probe construct (100μm wide), giving a high degree of confidence in the quantitative characterization of tissue forces.”

• Referring to the technique as “TFM” will inevitably result in confusion with Traction Force Microscopy, a very established technique in mechanobiology. A different abbreviation should be considered.

Thanks. We have revised the abbreviation to TiFM.

Reviewer 2 Advance Summary and Potential Significance to Field:
This is a concise paper but contains a new approach that enables quantification and manipulation of forces in living embryo. This sort of technical challenges are required and should be appreciated in the filed of developmental mechanobiology, especially if this instrument is applicable to other model animals' embryo.

Thank you. We are currently testing the system on zebrafish embryos which show promising results. We will present those in a follow-up report.

Reviewer 2 Comments for the Author:
Measurement and manipulation of physical forces that are applied on living tissues are very difficult technically and the development of such methodologies are certainly essential for the advancement of mechanobiology that uses living embryos. This paper by Chan et al. reports a new technology taking advantage of high sensitivity of force measurement by AFM, combined with sophisticated electro-engineering employing piezo and capacitor. This technique has been shown to enable not only measurement of minute forces generated in tissues of living embryos but also application of forces on tissues at any given time and place of embryos. This is an important step toward the further understanding of the physiological significance of forces during development.

This reviewer raises several minor points for improvement.

1. TFM is the abbreviation commonly used for traction force microscopy in the field of mechanobiology and thus it may confuse readers and the field. This reviewer suggests an alternative term, for example, TiFM.

Thanks. We have revised the abbreviation to TiFM.

2. In the summary, the word “non-invasive” may be too exaggerated. This may depend on how invasion is defined and it may be true if the method is compared to other harsh operations previously reported, but insertion of cantilever may be considered to be an invasion as it should cause some mechanical influence on the surrounding cells. Therefore, better wording, or elaboration of why this method is considered to be non-invasive, is recommended.

© 2023. Published by The Company of Biologists under the terms of the Creative Commons Attribution License (https://creativecommons.org/licenses/by/4.0/).

Thanks. We agree. Our technique, as a contact/insertion method, carries certain degree of invasiveness as compared to non-contact methods. However, while non-contact methods such as Brillouin microscopy are available to measure certain aspects of tissue mechanical properties, they are not able to directly measure tissue stresses. Our probe is less invasive in comparison to other available methods for stress measurement (e.g., embedding gels/droplets) for two main reasons: first, the probe has a minimized insertion footprint (a few microns thick, or in the case of using foils, 10-20microns thick); second, it is only needed to be inserted for the duration of measurement and can be retracted.

In the revision we added evaluation experiments to help readers judge the degree of invasiveness. In the new loading experiment added (Figure 3A and Movie S4), we show that the wound quickly closes and disappears within ~15 minutes after probe retraction, despite large deformation caused by the loading, no tissue tearing was observed.

The invasiveness is increased when foil is used. With foil, the open slit takes some time to heal. Taking advantage of our control of the piezo and subsequently the probe and foil, we actuated the end of the body axis to create different sized wounds. In the new Figure S3B we show results for the readers to evaluate the invasiveness of the foil. For a slit-like wound the tissue heals under 1hr, as the wound widens, healing becomes slower and not possible. These results suggest that temporary, thin wounds as generated in our experiments have a low level of long-term invasiveness, as the opening is small and can recover, compared to large implant methods.

In the revision we comprehensively described the invasiveness and put the technique in proper context. We also changed the text “non-invasive” to “minimally-invasive” in light of the evidence provided.

3. In Fig. 1E, the right most panel, the coloring of blue and red dots does not make sense. Are they reversed?

Thanks. The dots are indeed reversed. We corrected this error.

4. In all photos and movies, it is useful to indicate A (anterior) and P (posterior). For Fig. 2B, C, “Y (space)” is fine but additional labeling is helpful.

Labeling has been added as instructed throughout the figure panels.

Specifically, an A-P axis double ended arrow is now visible in relevant panels. For Fig. 2B,C, the Y axis has been relabelled as L to M (Lateral to Medial).

5. It is useful to provide the information of approximate depth of the force measurements (cantilever) from the tissue surface.

Depth information has been added for reported results. A detailed protocol on how to correctly obtain the depth information and the contact area with tissue is now provided.

Specifically, section 4.3 of materials and methods now includes: “*To determine the contact area between the tissue and the probe in order to calculate the stress (rather than just the total force), the insertion depth is measured by the Z positioning system of the microscope. Using the Zeiss Axio Observer as an example: first, the objective position is recorded from the Z-controller screen when the focus is on the surface of the tissue (e.g., endoderm for a dorsally mounted embryo such as in Fig. 1E); second, the objective is moved (lowered) to focus on the vicinity of the tissue layer of desired insertion depth (e.g., dorsal edge of the neural plate as in Fig. 1E); third, the probe (with or without foil) is inserted until the tip or edge of the foil is in focus at the desired depth, some minor adjustment of probe depth and/or focus might be performed for best focus and contrast, then the Z position of the objective is recorded again. Comparing the recorded objective Z positions yields the insertion depth. Using the insertion depth and known shapes of the probes and/or foils and the tissue, the tissue contact area can be estimated.*”

© 2023. Published by The Company of Biologists under the terms of the Creative Commons Attribution License (https://creativecommons.org/licenses/by/4.0/).

Second decision letter

MS ID#: DEVELOP/2022/201054

MS TITLE: Direct Force Measurement and Loading on Developing Tissues in Intact Avian Embryos

AUTHORS: Chon U Chan, Fengzhu Xiong, Arthur Michaut, Joana M. N. Vidigueira, Olivier Pourquie, and L Mahadevan

I have now received all the referees reports on the above manuscript, and have reached a decision. The referees' comments are appended below, or you can access them online: please go to BenchPress and click on the 'Manuscripts with Decisions' queue in the Author Area.

The overall evaluation is positive and we would like to published your manuscript in Development. Before we can proceed, can you please address the comments from Reviewer 2, namely to tone down the claims about other methods (see their comments).

Reviewer 1

Advance summary and potential significance to field

In this revised version Chan and colleagues have greatly clarified their manuscript. I think the technique is interesting and relevant to the developmental biology community but that some of the appreciations of the authors techniques are currently unnecessarily partial.

In the introductory section about techniques used to measure tissue mechanics the authors conclude that previous techniques had “limited success in measuring bulk tissue stresses in situ”. I do not agree with this subjective statement and I am not sure to see the benefit of such statement for the manuscript. Also, the authors list many techniques, including Brillouin microscopy, which is a promising new approach but that, to my understanding, does not currently unambiguously measure tissue mechanics. Also, since the list is rather exhaustive, microaspiration should be mentioned.

To legitimize the development of their new technique, the authors emphasize the “large deformation at the local embedding site” caused by other techniques. Yet the authors clearly describe how cells accumulate before the cantilever and become depleted after the cantilever (Fig1). I do not see how that differs from the effects of embedding droplets in the tissue. I think these repeated statements should be amended.

In the legend of figure 3, could the authors specify the sample sizes?

Comments for the author

In this revised version Chan and colleagues have greatly clarified their manuscript. I think the technique is interesting and relevant to the developmental biology community but that some of the appreciations of the authors techniques are currently unnecessarily partial.

In the introductory section about techniques used to measure tissue mechanics the authors conclude that previous techniques had “limited success in measuring bulk tissue stresses in situ”. I do not agree with this subjective statement and I am not sure to see the benefit of such statement for the manuscript. Also, the authors list many techniques, including Brillouin microscopy, which is a promising new approach but that, to my understanding, does not currently unambiguously measure tissue mechanics. Also, since the list is rather exhaustive, microaspiration should be mentioned.
To legitimize the development of their new technique, the authors emphasize the “large deformation at the local embedding site” caused by other techniques. Yet the authors clearly describe how cells accumulate before the cantilever and become depleted after the cantilever (Fig1). I do not see how that differs from the effects of embedding droplets in the tissue. I think these repeated statements should be amended.

© 2023. Published by The Company of Biologists under the terms of the Creative Commons Attribution License (https://creativecommons.org/licenses/by/4.0/).

In the legend of figure 3, could the authors specify the sample sizes?

Reviewer 2

Advance summary and potential significance to field

Mechanobiology in developmental systems has been hampered by the lack of technologies to directly estimate forces in living embryos of 3D structures in a non-invasive manner.
This methodology with the minimum invasion would provide a new tool to measure forces in living embryos.

Comments for the author

This reviewer thinks that the authors faithfully responded to the comments of the reviewers and judges that this paper is now acceptable.

Second revision

Author response to reviewers' comments

Reviewer 1 Advance summary and potential significance to field

In this revised version Chan and colleagues have greatly clarified their manuscript. I think the technique is interesting and relevant to the developmental biology community but that some of the appreciations of the authors techniques are currently unnecessarily partial.
Thanks very much for the evaluation and input. We made further changes to balance our views, highlighting the reviewer's input.

In the introductory section about techniques used to measure tissue mechanics, the authors conclude that previous techniques had "limited success in measuring bulk tissue stresses in situ". I do not agree with this subjective statement and I am not sure to see the benefit of such statement for the manuscript. Also, the authors list many techniques, including Brillouin microscopy, which is a promising new approach but that, to my understanding, does not currently unambiguously measure tissue mechanics. Also, since the list is rather exhaustive, microaspiration should be mentioned.
Thank you. We noted the limitation of Brillouin microscopy and added a reference to microaspiration technique (Kim et al., 2020). We also added more contexts to different techniques listed to be more informative for the readers. The paragraph now reads:
"A number of in vivo approaches have been developed to address this challenge in early embryos. These include classic embryology methods such as surgical cutting (Schoenwolf and Smith, 1990; Xiong et al., 2020) which allows inference of tissue mechanical interactions; microaspiration (Kim et al., 2020) which measures tissue mechanical properties; cantilever beams and fibres (Hara et al., 2013; Chevalier et al., 2016), and embedding gels (Zhou, Kim and Davidson, 2009) which measure tissue stresses; laser ablation (Hutson et al., 2009) which assesses tissue tension, among others (Campàs, 2016). Emerging (in the sense that they are more recently applied to early embryos) techniques incorporating precision engineering methods also show great promise, particularly in the measurement of tissue mechanical properties in intact embryos (or large explants). Examples include imaging-based methods such as optical coherence elastography (OCE) (Mulligan et al., 2016) and Brillouin microscopy (Prevedel et al., 2019) operating with certain mechanical models. Actuator-based approaches, such as magnetic droplets (Serwane et al., 2017), atomic force microscopy (AFM) (Barriga et al., 2018) and related microindenters (Marrese et al., 2020), and optically trapped nanoparticles (Dzementsei et al., 2018), offer direct mapping of the spatial-temporal mechanical heterogeneity of tissues and can also be used to introduce a controllable load to tissues in situ."

© 2023. Published by The Company of Biologists under the terms of the Creative Commons Attribution License (https://creativecommons.org/licenses/by/4.0/).

To legitimize the development of their new technique, the authors emphasize the ”large deformation at the local embedding site” caused by other techniques. Yet, the authors clearly describe how cells accumulate before the cantilever and become depleted after the cantilever (Fig1). I do not see how that differs from the effects of embedding droplets in the tissue. I think these repeated statements should be amended.
We agree. The presented method in some cases also creates large deformations. We clarified the related statements by acknowledging this limitation, specifying the actual advantage compared to embedding methods (thin and retractable), and highlighting that the presented method complements the embedding methods.
Related sentences now read:
In the introduction, we clarify that the comparison is being drawn with our own previous work, not generally all embedding methods: “Furthermore, as the size of the gels were relatively large (dozens of cell diameters) and stayed in the tissue for a long period of time (Xiong et al., 2020), the deformation induced by them at the local embedding site could alter the cell organization and tissue mechanics of the normal tissue environment, making tissue force quantification inaccurate. To minimize the tissue impact of force sensors is to use ultra-thin, retrievable probes, which reduces the size and duration of contact required for the measurements.”
In the results section, we note to readers that the stalling condition where cells accumulate and deplete is not a physiological condition and shows large scale deformation: “Cells are observed to accumulate anterior to the foil as the foil moves and eventually stalls (Movie S1). Posterior to the foil the cell density markedly reduces (Fig. 1F). Note that the stalling condition where large local tissue deformation has occurred does not represent the normal condition of the tissues but helps assess the force producing capacity of the tissue.”
End of the same paragraph: “(in contrast to large embedded sensors)” is removed.
In the discussion paragraph: “These features are advantageous as the tissues are measured more closely to their native state with smaller and shortened local deformations.”

In the legend of figure 3, could the authors specify the sample sizes?
Sample sizes have been specified as follows: “Loaded samples are under 150-200nN (n=4). 0nN indicates no load control on the TiFM (n=4). Incubator control includes samples not on the TiFM mounting environment (n=8), which develop faster”

Reviewer 2 Advance summary and potential significance to field

Mechanobiology in developmental systems has been hampered by the lack of technologies to directly estimate forces in living embryos of 3D structures in a non-invasive manner.
This methodology with the minimum invasion would provide a new tool to measure forces in living embryos.

Reviewer 2 Comments for the author

This reviewer thinks that the authors faithfully responded to the comments of the reviewers and judges that this paper is now acceptable.
Thanks very much for the evaluation and input.

Third decision letter

MS ID#: DEVELOP/2022/201054

MS TITLE: Direct Force Measurement and Loading on Developing Tissues in Intact Avian Embryos
AUTHORS: Chon U Chan, Fengzhu Xiong, Arthur Michaut, Joana M. N. Vidigueira, Olivier Pourquie, and L Mahadevan
ARTICLE TYPE: Techniques and Resources Report

I am happy to tell you that your manuscript has been accepted for publication in Development, pending our standard ethics checks.

© 2023. Published by The Company of Biologists under the terms of the Creative Commons Attribution License (https://creativecommons.org/licenses/by/4.0/).
